# “Why can’t you be normal?”: an urgent call for social and institutional inclusivity of neurodivergent scientists in STEM workplaces

**DOI:** 10.1128/mbio.00873-25

**Published:** 2025-11-19

**Authors:** John Houston Gardiner

**Affiliations:** 1 Huck Institute of the Life Sciences, Penn State University 8082 , University Park, Pennsylvania, USA; University of Wisconsin-Madison, Madison, Wisconsin, USA

**Keywords:** autism, neurodiversity, conduct of scientific research, research environments

## Abstract

The number of autistic people is growing rapidly. Among other traits, autism results in significantly more rational and objective thought patterns, and thus, neurodivergent students pursue education and employment in STEM fields at higher rates. However, autistic scientists experience discrimination and more career setbacks than neurotypical scientists. Despite the myth of scientific meritocracy, there are pervasive social expectations to “behave normally” and “fit in,” encouraging the camouflaging of autistic traits, which can contribute to anxiety, depression, and suicidality. The literature is conclusive: it is the obligation not of autistic scientists to change but of their managers, coworkers, administrators, and society to be more inclusive, accommodating, and tolerant of neurodivergent perspectives in work environments.

## PERSPECTIVE

## A DEMOGRAPHIC OF GROWING IMPORTANCE

Autism is a neurodevelopmental phenotype characterized by challenges with socialization (1). Officially classified as an intellectual disability (2), the term “neurodivergent” (antonym: “neurotypical”) is preferred to represent the diversity of phenotypes (3, 4) and redefine autism from a deficit-oriented disease to a neurologically based difference inextricably linked with one’s identity and perspective (5) (while more complicated, varied, and encompassing related or comorbid conditions such as ADHD or undiagnosed “twice exceptionality”, for the purposes of this perspective, neurodivergence is used interchangeably with autism as many of the findings and implications herein are broadly shared. Additionally, findings on the former diagnosis of Asperger’s syndrome (6) in some citations are combined with autism). For this reason, 87% of autism stakeholders prefer identity-first language (“autistic person”) over person-first language (“person with autism”) (7).

The number of autistic individuals is increasing rapidly; the CDC and other groups have identified a significant growth in prevalence (8) from 0.62% in 2002 (9) to almost 3% in 2020 (10). The cause of this rise is hotly debated between an improvement in awareness leading to increased diagnosis of a fixed number of cases or an actual increase in the incidence of autism (11). While the latter seems to be at least partially true (12), irrespective of the mechanism, that the number of diagnoses is growing means autistic individuals will only become more common in both classrooms and workplaces. It is thus that inclusivity of neurodivergence is an increasingly pressing matter.

Since autism typically results in a “consistent bias toward deliberative reasoning” (13) and enhanced rationality and objectivity (14), it should come as no surprise that autistic individuals are more likely than neurotypical individuals to seek education (15) and employment (16, 17) in STEM fields. Despite this, disabled individuals remain underrepresented in STEM (18), are less likely to persist in undergraduate education (19), experience bullying and discrimination as graduate students (20, 21), are less likely to be employed (22–26), have lower salaries and are less likely to get tenure (27, 28), are routinely dehumanized (29, 30), and, overall, experience “pervasive obstacles…rooted in bias and ableism…that [impact] professional relationships” (31). While there is a tendency in the literature to lump neurodiversity with other disabilities, which sometimes require very different accommodations resulting in a dearth of autism-specific research, various analyses corroborate these challenges as specifically pertaining to neurodivergence as well (32–36).

Due to the growing number of autistic people and their predilection for but significant difficulties in pursuing STEM fields, any problems faced by neurodivergent scientists or their employers are slated to increase in prevalence. It is therefore advisable for the scientific community to not just understand but practice more acceptance of neurodivergence. This perspective argues that neurodivergence leads to more objective and logical behaviors which, while potentially conflicting with neurotypical norms, need not be changed for properly functioning research environments and that demanding conformity of autistic scientists is actively harmful to their mental health, robbing the scientific community of their contributions.

## THINKING DELIBERATELY

Besides a predilection for rationality and objectivity (14), neurodivergent individuals exhibit altered speech and vocabularies (37–39), repetitive, calming behaviors (40, 41), difficulties with self-expression (42), tendencies for rigidity and literalism (43), and, sadly, higher rates of anxiety, depression, and suicide (44–46). To be sure, there are benefits to being autistic, but there have been unfortunately many attempts to “cure” autism by separating problematic and advantageous traits, particularly in the pre-2010 literature (47–50), although this may not be possible as many autistic people consider their autism integral to their worldview (5, 30).

Interestingly, neurodivergent individuals have difficulty with false-belief reasoning (51), implying impaired theory of mind (ToM) (52). However, recent studies have cast doubt on ToM as a causative mechanism of autism (53) and the literature is moving away from ToM- and empathy-deficit models of autism as oversimplified (54), negatively mischaracterizing (55), dehumanizing (56), and “zombie pseudoscience” (57).

Nevertheless, autism remains classically associated with an inability to recognize others’ emotions (58–61) and behaviors perceived as unempathetic or oblivious (62). This may be due to multiple factors: autism is known for inflexibility (43, 63), executive function deficits (64), and (reminiscent of false-belief challenges) poor imagination (65), even to the extent that autistic readers prefer nonfiction literature (66). While each of these traits is either debated (67, 68) or subtle, this may stem from neurodivergent individuals’ challenges with cognitive load, evidenced by increased memory and performance impairment than neurotypicals when distracted (69–73). It is even thought that a quintessential trait of autism—the inability to maintain eye contact—is a functional behavior to modulate cognitive demands during interlocution (74). Although debated (69) and while research does not often reveal *major* differences from neurotypicals, it is reasonable to conclude that, in complex, real-world (social) situations, the difficulties with flexibility, empathy, counterfactual reasoning, and imagination can combine and may exceed the available cognitive bandwidth (75), making it difficult or impossible for autistic people to put their acquired knowledge into practice (76). Incidentally, this may explain both the primary challenge of autism—social awkwardness—as well as the variability in the literature.

In essence, Grant et al. (77) summarized it neatly over two decades ago: “it appears that autistic children’s difficulties in passing standard false-belief tasks derive from the cognitive requirements of the tasks and not from a flawed conceptual understanding of belief.”

## A MATTER OF LIFE AND DEATH

Unfortunately, these divergent thought patterns of autistic individuals are not merely alternate strategies of perception, but often a source of friction with neurotypical peers, significantly impacting quality of life.

Social camouflaging, colloquially termed “masking,” is the deliberate suppression of autistic traits to behave “normally” (78) and includes widely varying and contextual behaviors such as forcing eye contact or suppressing “stimming” motions (i.e., fiddling with fidget spinners or pens, or bopping limbs) all the way to intensely self-policing language choices and obsessively studying and conforming to neurotypical expectations. Although initially posited to explain the underdiagnosis of autism in females (79) (while camouflaging is more common among autistic females due to gendered socialization, it should not be mistaken as an exclusively female trait [80, 81] as it is also present in males and most prominent in nonbinary individuals [82, 83]), camouflaging research has only recently taken off, with PubMed indicating ~75% of papers having been published in the last 5 years. Camouflaging is even observed as being similar to how racial minorities alter their behavior to “perform” whiteness and fit in with the dominant culture (84). While there are, of course, many confounding factors to this comparison, it at least implies a common, intersectional conflict originating from shared in-group/out-group dynamics.

Camouflaging is often motivated by feelings of social outcast and a pressure to conform, and frequently results in stress, exhaustion, and an eroded sense of self, anxiety, depression, “autistic burnout,” suicidal tendencies, and overall lower quality of life (79, 80, 82, 85–100). As superbly and succinctly examined by both Mandy (101) and Hull and colleagues (83) (upon first reading their paper, this author burst into tears and ugly-cried for 90 minutes from the validation of lifelong issues), camouflaging takes extreme effort to monitor and deliberately suppress one’s natural tendencies, but represents a catch-22 wherein camouflaging may be necessary to ameliorate bullying, gain acceptance, have healthy social relationships, or (this author’s addition) maintain employment. Worse still, it seems that classically autistic comorbidities such as ADHD or oppositional defiant disorder transform into anxiety and depression as neurodivergent individuals age, learn self-control, and proceed to mask their behaviors (83).

In effect, neurodivergent people are not given the option of having anxiety or depression, but may instead only choose how it is administered: behave authentically but be socially isolated for their differences, or conform to neurotypical expectations and suffer the problems associated with self-suppression. This results in a minority tax, wherein, as is the case for other minority groups, neurodivergent scientists are expected to deal with all the typical hardships of graduate school and tenure-track positions, and then some. It is thusly that efforts to modify an autistic person’s behavior have been likened to the resoundingly unethical gay conversion therapy (102).

##  OPINION/HYPOTHESIS: TOWARDS A MORE INCLUSIVE SCIENCE 

A major difficulty with autism is the elusiveness of the symptoms. In contrast to physical disabilities with tangible needs (e.g., ramps for wheelchair access or ample and appropriate seating) and which are made less arduous with social acceptance or institutional flexibility (e.g., expedience in addressing those needs), neurodivergence is treated largely with social acceptance and institutional flexibility (in this author’s experience, the single most common accommodation requested by neurodivergent students is “patience,” which is understandably difficult to make policy). Further, being a largely invisible disability, it is difficult to parse which aspects of autistic behavior are merely personality and which are unchangeable disability traits. While musing whether this is even possible is beyond the scope of this perspective, in this author’s experience, the current STEM culture is much more inclined to statements like “why can’t you be normal?” and “you should learn to behave like a normal person,” (actual sentences said to this author).

While there is almost no research on the subject, it is easily inferred that autistic individuals are predisposed to interpersonal conflicts in the workplace. Besides the robust findings that autistic individuals are often more logical, rational, objective, and unbiased (13, 14, 103–109), there is also evidence that they prioritize common moral norms (61, 62, 110, 111), preferentially utilize utilitarian ethics (60), are less susceptible to the bystander effect, less complacent with the status quo, and more likely to intervene (even if intervention is unpopular) (112), and may even possess “super morality,” behaving more morally than neurotypicals (113, 114) and being more distressed by immorality (115). Furthermore, autistic individuals are often mistaken as unempathetic (62), have more cerebral, deadpan, and self-deprecating humor (116–118), and possess low respect for nonspecific virtues such as loyalty and sanctity (110, 119). It should not be surprising that the combination of these traits can often be a recipe for workplace disaster.

In trying to improve acceptance and productivity of neurodivergent employees, Seitz and Smith identified five strategies: managers should be compassionate, flexible, motivated by policy, give explicit instructions, and provide ongoing social support (120). A recent roundtable of autistic scientists echoed these and added that autistic researchers should be treated as equals, that their perspectives be appreciated and validated, and that autism is not something to be “fixed” (121).

While no list could be exhaustive, this author would like to add some salient points. First, the role of patience cannot be understated; given that autistic people exhibit slower, more deliberate reasoning, must decode social subtleties, and are more likely to be intellectually disabled (122), simply accepting that it might take longer for autistic individuals to understand something, or that they go about understanding things in different ways, would be extremely helpful. Additionally, allowing neurodivergent individuals to behave naturally (rather than expecting conformity) and being open to different perspectives, workflows, learning strategies, humor styles, and limitations would significantly benefit the mental health of autistic (5, 123–126) and neurotypical coworkers alike.

Most importantly, all these recommendations should be reflected at the administrative level (120), as rigid policies or a bureaucratic unwillingness to accommodate neurodivergence can undo the work of an accepting lab. Administrators and managers should be empowered (and encouraged through holistic funding or reward systems) to make individual, contextually-derived exceptions directly at the point of conflict, especially because targeted accommodations would not impose a broader institutional burden. As noted by Belek (76), “many autistic people regularly work to develop an expertise in social etiquette, while remaining stubbornly inept in putting this theoretical expertise into practical usage.” Ergo, there are at least some things for which autistic individuals simply cannot meet neurotypical expectations, and one would be loath to hold all to standards which only some can meet.

An excellent example of how contextually-derived accommodations might work can be found in autistic individuals’ hypersensitivity to stimuli (127), particularly sounds (128). Autism is comorbid with misophonia, a neurological condition wherein very specific sounds (such as chewing noises or a certain musical beat) elicit intense emotional responses of anger or distress (129). While enforcing a university-wide ban on chewing noises would be less than practical and navigating differing music tastes is part of the territory of coworker relationships, holding an autistic scientist to the same standards as a neurotypical one by forcing them to “suck it up” and deal with distracting or triggering sounds would put an asymmetric burden on neurodivergent employees. In these examples, requiring that eating be conducted in break rooms or that headphones be worn when in the lab space would not significantly encumber neurotypical workers, but would make a humongous difference for the neurodivergent scientist’s productivity and quality of life. Whereas global policies to accommodate the whole spectrum of neurodivergence might be impossible to codify, this does not equate to neurodivergent workers being undeserving of accommodations made specific to each situation.

It is important to note that this author is not advising policy anarchy, jettisoning all convention at an autistic person’s discretion the moment they walk in the door. However, it is undeniable that a strict interpretation of all institutional policies in all situations is not expressly necessary for a functional workplace (at a previous position, it was once made clear to this author that continued employment was dependent on walking less flamboyantly through hallways), and that, sometimes, a degree of patience or compassion would go very far to improving working environments for all involved. In many cases, simple misunderstandings are far too quickly escalated and result in (or demand) camouflaging, directly harming the neurodivergent worker (78–101, 123). When an autistic individual is expected to follow a rule seen as arbitrary, illogical, or subjective, the tendencies for rule-following and social rigidity (43) conflict with the hyper-rationality (13, 14, 104–108) and intolerance of uncertainty (40, 130–132), causing a double bind which is, in this author’s direct experience, existentially debilitating. This is neither compassionate nor necessary for the production of good research or maintenance of amicable coworker relationships.

As observed by one neurodivergent scientist: “[i]sn’t it ironic how inflexible neurotypical society can be, despite the traditional focus on autistic inflexibility?” (121).

## CONCLUSION

Employing neurodivergent individuals does not burden organizations (28), neurodivergent employees are more likely to report organizational inefficiencies (112), and science would do well to harness autism’s predilection for unbiased, rational instincts. This can only be achieved by managers, administrators, and coworkers accepting the limitations of autistic colleagues through empathy, flexibility, and targeted accommodations.

Historically, early disclosure of autism diagnosis was considered advisable as it sets realistic expectations, increases understanding, ameliorates the need for camouflaging, and improves personal relationships (123, 125, 126). Indeed, many of the suggestions offered herein are dependent on full knowledge by both parties. However, the current U.S. political climate is clashing with established science (133), enacting policies toward autistic people which are “terrifying,” “dehumanizing,” “dangerous,” and “eugenic” (134–137). That the current U.S. administration is unashamedly emulating the first steps of Nazi Germany’s genocides by establishing a registry of neurodivergent individuals is “gravely [concerning]” (138–140). It should, therefore, be understood that disclosure of or formalizing a diagnosis in the present climate may be expressly dangerous, and that–even in the absence of medical proof–flexibility, accommodations, and patience should be offered freely and anyway by managers and administratorss to anyone in need.

Autistic students pursue STEM careers at significantly higher rates. However, despite the myth of meritocracy, STEM majors are not more accepting of autism (141); neurodivergent individuals face ubiquitous obstacles to achievement and pervasive gaslighting that autism is a disabling defect which must be corrected (5, 30, 80, 121, 142–144), particularly as productivity shifts from more regimented classrooms to more social workplaces. Research unequivocally demonstrates that asking autistic individuals to “behave normally” increases anxiety, depression, and suicidality (78–101, 123). Autism is not a deficit but a different, equally-valid perspective which should be welcomed and appreciated. The scientific literature is conclusive: it is society which must change and not its autistic members (20, 29, 30, 102, 145–149).

In an interview with *City Cast Pittsburgh*, disability advocate Alisa Grishman probably put it best where from the challenges of disability originate: “People use the phrases ‘confined to a wheelchair’ or ‘wheelchair bound,’ [but that] makes a wheelchair sound like an imprisoning thing….In a world that had great sidewalks, sliding doors, ramps, I wouldn’t be disabled, I’d be able to get anywhere just the same as someone who’s currently able-bodied can do now. It’s the world that creates the disability, not my chair” (150).
